# What motivates public collaborators to become and stay involved in health research?

**DOI:** 10.1186/s40900-024-00555-5

**Published:** 2024-02-12

**Authors:** Toril Beate Røssvoll, Kristin Liabo, Tove Aminda Hanssen, Jan H. Rosenvinge, Elisabeth Sundkvist, Gunn Pettersen

**Affiliations:** 1 Department of Health and Care Sciences, Faculty of Health Sciences, UiT The Arctic University of Norway, 9037 Tromsø, Norway; 2 College of Medicine and Health, PenCLAHRC Patient and Public Involvement Team, University of Exeter Medical School, Exeter, UK; 3 Centre for Shared Decision Making, University Hospital of North Norway, Tromsø, Norway

**Keywords:** Patient and public involvement, Public collaborator, Qualitative research, Reflexive thematic analysis, Maintaining motivation

## Abstract

**Background:**

People with lived experience of health and illness are increasingly being involved in research. Knowing what creates interest in becoming involved in health research may help identify appropriate ways of facilitating meaningful involvement. The study aimed to investigate why people became public collaborators in health research and what helped sustain their commitment to staying involved.

**Methods:**

Semistructured individual qualitative interviews were conducted with 11 Norwegian public collaborators recruited from patient organisations. To enhance the quality and relevance of this study, three public collaborators were involved in framing the study and in the data analysis. One of them is a coauthor of this paper. The interviews were analysed through reflexive thematic analysis, and two themes were generated.

**Results:**

The first theme, ‘research as a vehicle to impact’ showed how interest in becoming involved in research was founded on the possibility of impacting healthcare through research. Other inspiring factors were how they appraised the relevance of the research, in addition to the public collaborators’ own sense of moral duty to advocate for research related to their own as well as others, illnesses or diseases. The second theme, ‘‘Acknowledgement and accessibility’, framed how the participants perceived appreciation of experiential knowledge as crucial for maintaining motivation in their role as public collaborators. Other promoters of sustained involvement presented were training for both public collaborators and researchers, adequate allowance as a means for visualising and valuing PPI, and accessible language.

**Conclusions:**

This study contributes to the understanding of how to facilitate meaningful and sustainable PPI, which requires a safe space for collaboration and attention to accessibility. Facilitating meaningful involvement may, in turn, increase the potential impact and sustainability of PPI.

**Supplementary Information:**

The online version contains supplementary material available at 10.1186/s40900-024-00555-5.

## Abstract (plain language summary)

It has been more common to involve people with lived experiences of health and illness to work with researchers as members of their teams. There is a general assumption that involvement may increase the relevance and impact of research, prompting research funders to require an outline of involvement strategies to obtain research funding. Understanding why people become involved in research may be helpful to improve how researchers and people with lived experience can work together in a good way. In this qualitative study, we interviewed 11 people with experience from involvement, based on their experiential knowledge as patients or next-of-kin, in health research. Three public collaborators were involved in the analysis workshops, and the interviews were analysed using reflexive thematic analysis. Two themes were developed. The first theme, ‘Research as a vehicle to impact’ showed how interest in involvement was triggered by the possibility of impacting health care services through research. The second theme, ‘Acknowledgement and accessibility’, framed the value of appreciation of experiential knowledge, besides the importance of making the research arena accessible for the public in terms of training, payment, and comprehensible language. Meaningful PPI creates a foundation for sustainable recruitment of public collaborators and raises the chances for PPI to have an impact.

**Supplementary Information:**

The online version contains supplementary material available at 10.1186/s40900-024-00555-5.

## Introduction

Patient and public involvement in health research (PPI) is a vital part of democratising knowledge production. There is a general assumption that PPI may enhance the relevance and clinical usefulness of health research. International and national funding calls now ask researchers to outline how patients or members of the public will be involved in the research process. Knowledge about what creates interest in becoming and motivation to stay involved can help researchers plan PPI better by identifying good and poor examples and drawing on previous experiences of involvement [1]. PPI typically means ‘an active partnership between patients, carers, and members of the public with researchers that influences and shapes research’ [2]. In this study, we adopt the term ‘public collaborator’ (PC) when referring to members of the public, patients, or next-of-kin bringing in both their own and others’ lived experience and perspectives’ in research [3].

Considerable attention has been given to understanding what PPI can bring to research, people involved, and how it impacts researchers [4–7]. These areas of knowledge are still in development, including how PPI can be impactful and how it can best be evaluated. As with any initiative, PPI is likely to be more impactful, including providing positive outcomes for the people involved, if implemented in a good way [8, 9]. Consequently, there is growing interest in exploring how to conduct PPI in meaningful ways that enhance the impact [3, 10].

Providing individual feedback to PCs has been shown to increase the impact of PPI and their motivation to remain involved [10, 11]. Taking experiential knowledge seriously and sharing power equitably is also recognised as critical elements for success [3, 12, 13]. Furthermore, the importance of PPI being well-planned and supported within the organisation is another reported success factor [10, 14, 15]. This includes establishing systems for compensating people for their time and travel, and ensuring that communication is tailored and accurate [16].

However, this study extends previous research by exploring people’s reasons for becoming involved in health research and their motivation for staying involved. Such knowledge can inform strategies to optimise the planning and implementation of PPI, and thus increasing its potential impact.

This study aimed to address the knowledge required to carry out successful planning of PPI by investigating PPI in health research from the perspective of public collaborators, guided by two research questions: (1) Why are people interested in becoming public collaborators in health research? and (2) what motivates public collaborators to continue their involvement?

## Methods

### Design and PPI in this study

This paper analysed qualitative data generated through individual semistructured interviews, as this approach is suitable to inform about PCs experiences and views of PPI [17].

The overall purpose of PPI in this study was to enhance the relevance and quality of the research. Three PCs were involved in the study, along with four academic researchers and a PhD student. The team had a variety of PPI experience from different research fields ranging from PPI facilitator, PPI researcher and public collaborator. The PCs contributed to the development and piloting of the interview guide and were involved in the data analysis, and one of them is a coauthor of this paper. Further description can be found in the reporting checklist in Additional file 1, following Guidance for Reporting Involvement of Patients and Public (GRIPP2-SF) [15].

### Recruitment

A purposeful sampling approach was used to recruit participants > 18 years old with experience as PCs with experience in a variety of research fields and PPI roles. No other inclusion criteria were set. A brief outline of the study was sent to the Norwegian Cancer Society and the Norwegian Federation of Organisations of Disabled People. These two umbrella organisations for people with disabilities and chronic or severe diseases in Norway keep track of PPI experience in their member organisations. Additional potential participants were identified by the PCs in this study through their patient organisation network. This resulted in a list of potential study participants, and the first author sent an email invitation to people on this list. One of the participants later shared the invitation with a peer.

Eleven people (eight women and three men) accepted the invitation and signed an informed consent statement, recruited from eight different patient organisations. These patient organisations are working to safeguard and promote the interests of people affected by various conditions, including cancer, neurodiversity, and other chronic diseases. They also provide counselling services, organise networking activities, and share research and specialist knowledge about their members’ conditions.

### Procedure

We developed a semistructured interview guide based on the research questions and relevant literature. Topics in the interview guide included motivations for getting involved in research, what influenced their continued engagement, and suggested areas for improvement. A pilot interview was conducted with one of the PCs in this study, and minor changes were made to clarify the meaning and encourage a fuller response.

The first author conducted all interviews online. Participants’ age, geographical location, name of patient association, and position of trust (if any) were obtained at the start of the interview. The interviews lasted 30–60 min. All participants were familiar with the use of virtual platforms for meetings, and the use of video ensured a visual connection between the interviewee and interviewer. The use of M365 Teams was considered appropriate as virtual interviews enabled access to participants from different geographical locations in a cost-effective way.

### Data analysis

We used a reflexive thematic data analytic approach appropriate to explore interview data and develop themes across the dataset [18, 19]. Reflexive thematic analysis is not affiliated with specific theoretical concepts or philosophical positioning [18]. It was thus considered suitable for this study as the involvement of PCs requires a flexible approach. For training purposes, the research team examined the six phases of reflexive thematic analysis at the start of the process, and the PCs signalled that they would prefer to be involved when discussing preliminary themes. The first author transcribed the interviews and became familiar with the data through repeated readings (phase 1). To capture the diversity of meaning within the dataset, the transcribed interviews were coded (phase 2), and the codes were clustered into initial themes (phase 3). The software NVivo 12 (QSR International) assisted in the process of coding and developing themes. Three of the authors (TBR, ES and GP), together with the PCs, examined participant demographics and the initial themes in two 3-h analysis workshops to develop and review the themes (phase 4). Among the preliminary findings discussed at the workshops were training, recruitment, and the increased need for people to become PCs. The workshops provided an arena for reflecting on the assumptions each of us brought into the dialogue, which involved engaging more deeply with the data and looking for the meanings and patterns in the data. Naming and writing up the themes (phases 5 and 6) involved a recursive process of refining the analysis.

### Ethical considerations

The study was approved by the Norwegian Agency for Shared Services in Education and Research, ref. nr. 292640, and followed the principles of the Helsinki Declaration in terms of informed consent, the option of unconditional withdrawal and anonymisation. Data storage was secured and adhered to the information security and data privacy management systems at UiT The Arctic University of Norway. The Regional Committee for Medical and Health Research Ethics (REK Nord) preassessed the study not to be subject to presentation. Pseudonyms were used for quotations illustrating the results to maintain the participants’ anonymity. Due to ethical considerations concerning involvement in recruitment and data analysis, the PCs did not get insight into the names of participants accepting the invitation.

## Results

### Participant demographics

The participants were geographically located across all five health regions in Norway, and the average age of the participants was 65 years (55–77 years range). They had experience-based knowledge as patients (n = 8), next-of-kin (n = 2), or a combination of the two (n = 1). Additionally, four participants had research experience related to their careers, including two PhDs, although not within health research. All participants held positions of trust in patient organisations or had previously done so, either as board members, chairpersons, volunteers who offered peer support through their own experience, or as part of a user council in hospital governance.

All the participants had been PCs in health research, ranging from one study (n = 4) to several (n = 7), including basic science projects, health service research, and implementation research related to their experiential knowledge of a particular condition or associated health issues. Together, they covered ten research units, mostly affiliated with universities and university hospitals. Most of the participants had experience being involved in projects that had already been developed by the time they joined, meaning they were not involved in codesign but invited at a later stage once the studies were designed by researchers. Some of the participants had been involved in research for several years and shared experience from the time before and after PPI became a prerequisite for health research funding applications in Norway. The reflexive thematic analysis of interviews generated two themes, presented below.

### Research as a vehicle to impact

The participants explained their interest in becoming PCs by their desire to learn as well as a genuine interest in contributing to research to improve health care services. Their own, as well as others, experiences with a diagnosis or illness created a personal foundation and engagement to partake in research as PCs and to address issues they found important but not typically addressed by research. Leah explained it as follows:

This project is revolutionary in the way that it can help a group of patients who previously were not offered any kind of treatment. I think it’s important and exciting to be part of it, also because I’ve got personal motivation for the project to succeed. / Leah (in her 50s, patient PC, >2 projects).

The participants described a strong link between their experience of the relevance of the research topic and their interest in becoming involved. The importance of PCs making a choice to become involved in projects that are close to their hearts and where they see their experiential knowledge can be of use was underlined. Involvement in already developed projects was triggered by the enthusiasm shown by the researcher presenting the project as well as how the PCs considered its importance, as expressed by Dina:

I heard the leader of the project at a lecture earlier, and I got very engaged! I was engaged and motivated because I believed in the concept {home-based diagnostic tool}. Therefore, when I was asked to join, I found it just great! One of the things I thought about was that this might change our future possibilities! / Dina (in her 60s, patient PC, one project).

Filip explained a moral responsibility to ‘step up’ for research related to their own disease or illness as a reason to get involved:

I generally encourage our members to become public collaborators because it is important for research considering our diagnosis that we step up and help, and it is also a requirement to involve public collaborators. / Filip (in his 60s, patient PC, >2 projects).

Also related to their roles as chairperson of patient organisations was the experience that recruitment of PCs was more challenging than recruitment of volunteers for other activities, such as peer support for patients with similar conditions or a public collaborator role within hospital governance. The participants reflected on how this could be due to people’s limited knowledge of PPI and research in general. Informing members about research and providing PPI training was described as a central task for the patient organisations, aiming for more members of their patient community to develop an interest in getting involved in research.

### Acknowledgements and accessibility

Preparing for and speaking their mind at meetings were described as valuable for shaping their role as PCs. Confidence in the role as a PC was motivating, as described here:

I feel like it becomes more professional, and you get more involved if you let yourself be involved. Because if I don’t make up my own opinion and if the project does not interest me that much, I can’t contribute in a good way. When there is a project that engages me and I find important, I put a lot more time into preparation. / Nathalie (in her 50s, patient PC, >2 projects).

Shaping the role of the public collaborator was explained as a mutual responsibility. The researcher’s ability to create a welcoming space was described as encompassing active appreciation of experiential knowledge besides facilitating clarity on the role of the public collaborator. Researchers’ feedback, acknowledgements, and active listening to the input from PCs were expressed as highly valuable for stimulating and inspiring. Arranging a visit to the research lab or being invited to a research group meeting with thorough and accessible information about the project was described as crucial to the start of a project.

Everybody {in the project} has been very positive about the public collaborators being involved {…}. The dialogue with the project management has been very good, and relevant information has been provided. I felt welcomed, and meetings were facilitated, and then you get motivated to contribute./ Leah

In contrast, not being contacted or invited for further discussion after having provided a letter of support or being recruited as a public collaborator was described as demotivating and a missed opportunity for both the researcher and the public collaborator. This could lead to withdrawal from the project or not being willing to be involved next time invited. From the experience of being part of a group of PCs, Adrian explains how they had to take initiative to avoid the involvement staying tokenistic:

We were just asked for a letter of support for the research project, and then we didn’t hear anything more from the researchers. Eventually, we were asking to be part of the project ourselves./ Adrian (in his 70s, next-of-kin PC, >2 projects).

Some of the participants reported having been involved in research projects without a plan for how this would be organised and facilitated. Overall, they noted that there had been a change for the better over the last three or four years. They felt that the researchers’ interest in PPI and attitude towards experiential knowledge had improved and therefore enabled change in terms of how PCs are treated.

In the beginning, it seemed to be difficult for the researchers to figure out how to handle this user involvement. Back then, there was no experience with this among the users either. (…) It’s gotten a lot better the last three or four years./ Adrian

The participants, particularly those with professional research experience in basic science, described how their previous knowledge of research influenced not only what projects they got involved in but also how they could perform their role as PCs.

Providing training in PPI can help people make a well-informed choice and establish their role. Some of the participants reported being invited as PCs after taking part in training, assuming that their PPI training was the reason why they were invited. Others experienced learning along the way, and in some cases, training was offered after the involvement had started. Training was explained as useful for being introduced to the research process and partaking in reflections on the role of experiential knowledge in research. Some of the participants indicated the advantage of researchers and potential PCs taking part in the same training, pointing out the need for researchers to prepare for collaborating with PCs.

I do not think you have to take up a particular education to be involved in research, although I find it important to know a little bit of the research process, especially some knowledge about where we can contribute and what is expected of you.” / Leah

The participants, who belonged to smaller patient organisations, explained how they depended on their umbrella organisation to provide PPI training and suggested that both research institutions and patient organisations should be equally responsible for the provision of training. From their experience as chairpersons of patient organisations, they reported that researchers increasingly requested suggestions for persons to be involved in research after PPI became mandatory in research funding applications. The role of patient organisations related to PPI training was described as important to provide candidates for relevant research projects.

Compensation for the PCs time use in terms of payment was emphasised as an aspect of making PPI accessible. Apart from recent years, participants reported that payment for involvement had not been discussed in the research group they were working with, and most of them had not received compensation for their research involvement. Although they expressed that payment was not a key motivator for them personally, they underlined allowance for PCs as an important principle, as expressed by Leah:

I think it’s not the money itself that’s important for users. I think that it has something to do with you feeling sort of… that your efforts matter. Because it’s our time too, even though many of us are not in permanent jobs, we spend time and energy on it. We should get something out of it; it goes without saying, although many may forget about it.

Including PPI in the research budget and providing payment for the time used for preparations and meetings were described as crucial initiatives making PPI more visible, as proof of valuing the involvement of PCs, and as contributing to equality between the members of a research group.

Practical support was highlighted as a particular need that was not always met. This could include researchers being available by phone or email for PCs outside of meetings besides providing information about the project status outside of meetings. Most of the participants in this study were not prevented from taking part in meetings during the working week and office hours. However, they underlined that mutual flexibility with respect to the participants’ working hours, family situations, and health issues could improve accessibility to research involvement, as such flexibility would enable more people to find time to be involved. The participants experienced that the meeting times were mostly adapted to the workday of the researchers, to which the PCs were expected to adjust. This is how Nathalie expressed it:

The meeting times have been more adapted to them than to me, although they’ve always asked, “Is the time fine with you?” And since I’ve had a job that has allowed me to do so, it’s been fine with me.

Accessibility also included keeping the language at a comprehensible level, which was stressed as vital to fostering continued motivation. Taking time to explain, aided by providing a list of words and abbreviations most commonly used in that scientific field, was expressed as facilitating meaningful discussions at research group meetings. The relationship between accessible language and its impact on dissemination is demonstrated by Filip’s explanation of how accessible language may impact the dissemination of research findings:

Just how they manage to pass it on, how they probably will communicate it, and how we as users can pass it on to other users. For example, based on our last meeting, I’ve got an idea of topics that we can raise at member meetings. We can invite the researchers to a member meeting so they can talk about their research there.

## Discussion

This study aimed to investigate people’s interest in becoming involved in health research and what helped sustain their motivation for continued involvement. The findings describe key considerations for people contemplating involvement in health research, as well as important aspects for achieving meaningful and sustainable PPI.

The first theme shows that people’s general interest in contributing to improving health care services and their perceived relevance of the research topic considering their experiential knowledge form an interest in becoming a public collaborator. These findings align with previous research also reporting on scientific curiosity and a belief that the study they were involved in would help other patients and their families, which is of importance [1, 5, 20, 21]. This also demonstrates the importance of providing accessible information in the planning phase of PPI so potential public collaborators can make a conscious choice about what project to be involved in. It is stated that involvement based on one’s own interests makes PPI more meaningful, which can benefit both the people involved and the research [12]. Most likely, a mixture of rationales underpins the motivation for people to become involved, and it is pivotal to address and manage the expectations linked to the motivation when recruiting. Addressing challenges through clarification of roles and expectations has been repeatedly acknowledged [12, 13]. The findings in our study underline role clarification as a means for avoiding tokenistic involvement.

The participants in this study had experience with civic commitments other than PPI, as they held positions of trust in patient organisations or had previously done so. The findings considering the training and recruitment of PCs, seem to be founded on their experience with civic commitments, which aligns with other research indicating that the impact on the research process is based on engagement on a broad scale and professional competence [22]. Interest in becoming involved in research is also reported to be seen as a continuation of involvement in other areas of civic engagement [21, 22].

The second theme elaborates on the value of acknowledging experiential knowledge and the factors of importance in making PPI accessible. The importance of appreciating experiential knowledge to gain confidence in the role of public collaborator is also underlined. These findings add to previous research showing that the way experiential knowledge is viewed and welcomed is crucial for PPI to be a positive experience for all involved and for PPI to have an impact [3, 16]. Our study brings additional insight to this by underlining the role of the umbrella organisations in the provision of training, which is also pointed out as an aid to prepare for the recruitment of PCs.

Previous research has underscored the importance of providing thorough information about the project at the outset of a collaboration and offering education and training for both PCs and researchers to achieve a comprehensive understanding of PPI [11, 23–25]. This study expands upon these findings by providing knowledge of the role of training and information in making the research arena accessible. Effective communication skills and the use of clear, comprehensible language are identified as key factors that facilitate ongoing involvement [11, 21]. These elements are integral to accessibility, which is crucial for democratising knowledge production and involving people from a range of socio-economic backgrounds.

### Strengths and limitations

This study’s strength is its originality as one of the few studies focusing on the experience of PCs in varied health research in Norway. Although small with respect to the number of participants, they brought in a range of perspectives based on various experiences with PPI from several Norwegian research institutions.

The involvement of PCs is part of a long-term involvement, and for this study, it provided richer discussions in the research team with several viewpoints represented. Contributions to recruitment and analysis enhanced the relevance and quality of the study. ES’ involvement in the writing process helped to nuance and validate the reporting, especially the description of patient and public involvement in this study.

One concern is the recruitment procedure; people who sign up for this kind of study may hold a positive attitude and opinion about PPI in health research. This potential bias, however, can serve as an incentive for future research to incorporate a wider array of opinions about PPI when exploring the PPI experience from the perspective of PCs. Moreover, in the interest of health equity, it is important to consider diversity in the socio-economic backgrounds of both PCs and participants in PPI studies to ensure a comprehensive understanding of the impact of PPI across different societal groups.

## Conclusion

This study contributes to existing PPI knowledge by providing insight into the experiences and views of PCs on PPI in health research in Norway. The essential elements for planning meaningful involvement identified in this study include creating interest in research and fostering ongoing involvement. Acknowledging experiential knowledge and ensuring that knowledge production is accessible to the public are crucial. Accessibility can be improved through appropriate budgeting for PPI, the provision of training, and the use of comprehensible language, all of which can facilitate conditions for sustained involvement.

### Supplementary Information

**Additional file 1**. GRIPP2-short form.

## Data Availability

The qualitative interview data (in Norwegian) generated and analysed during this study are not publicly available.

## Abbreviations

PPI: Patient and public involvement
PC: Public collaborator
