# Supplementary material for: What motivates public collaborators to become and stay involved in health research?
Source: Res Involv Engagem. 2024 Feb 12;10:24. doi: 10.1186/s40900-024-00555-5 (PMC10863223; doi:10.1186/s40900-024-00555-5)
Supplement: Supplementary file 1 — Additional file 1. GRIPP2-short form. [file 40900_2024_555_MOESM1_ESM.docx]

# Additional file 1. GRIPP2-Short form

| Section and topic | Item | Reported on page no. |
| --- | --- | --- |
| 1: Aim | *Report the aim of PPI in the study.*  The overall purpose of PPI in this study was to enhance the relevance and quality of the research by bringing in experiential knowledge to inform it. Since the topic is PPI, it was paramount to design the process with PPI. | Page 4. Methods / Design and PPI in this study. |
| 2: Methods | *Provide a clear description of the methods used for PPI in the study.*  Three public collaborators were involved in the research team, along with four academic researchers and a PhD student. The public collaborators contributed to recruitment, the development and piloting of the interview guide, and took part in analysis workshops. One is a coauthor of this article. | Page 4, 5 & 6. Methods / Design and PPI in this study / Recruitment / Analysis. |
| 3: Study results | *Outcomes – report the results of PPI in the study, including both positive and negative outcomes.*  Public collaborators involvement in recruitment, analysis, and reporting enhanced the relevance and quality of the study. The discussions got richer, with several viewpoints represented. ES’ involvement in the writing process helped to nuance and validate the description of patient and public involvement in this study. | Page 15. Discussion / Strengths and limitations. |
| 4: Discussion and conclusions | *Outcomes – comment on the extent to which PPI influenced the study overall. Describe positive and negative effects.*  We shared a sense of purpose in the project group. Planning and experiencing, along with researching PPI, were instructive and inspiring. | N/A |
| 5: Reflections / critical perspective | *Comment critically on the study, reflecting on the things that went well and those that did not, so others can learn from this experience.*  The level of involvement in the different phases of the project was balanced according to budget and timeframes. For instance, reading interview transcripts was not prioritised, although the public collaborators expressed interest in this. Involvement in data analysis was arranged as workshops to best align with the budget and timeframes.  Challenges due to widespread geographical locations were addressed and solved in a pragmatic manner. Virtual and hybrid meetings have been inspiring, motivating, and a place for all members to bring relevant issues to the table. However, a physical encounter early in the collaboration would have been preferred. It took some time at the start of collaboration to sort out the practical arrangements for payment. The patience shown by public collaborators was appreciated. | N/A |
